# Molecular Subtyping of Human Rhinovirus in Children from Three Sub-Saharan African Countries

**DOI:** 10.1128/JCM.00723-19

**Published:** 2019-08-26

**Authors:** Vicky L. Baillie, David P. Moore, Azwifarwi Mathunjwa, Palesa Morailane, Eric A. F. Simões, Shabir A. Madhi

**Affiliations:** a Department of Science and Technology/National Research Foundation: Vaccine Preventable Diseases Chair, Medical Research Council: Respiratory and Meningeal Pathogens Research Unit, University of the Witwatersrand, Johannesburg, South Africa; b University of Colorado School of Medicine and Colorado School of Public Health, Aurora, Colorado, USA; University of North Carolina School of Medicine

**Keywords:** molecular epidemiology, pneumonia, rhinovirus, viral load

## Abstract

The pathogenesis of human rhinovirus (HRV) during severe respiratory disease remains undefined; thus, we aimed to explore the relationship between the HRV molecular subtyping results obtained during severe and asymptomatic childhood infections. Nasopharyngeal/oropharyngeal swabs from children (1 to 59 months of age) hospitalized with pneumonia and from age-frequency-matched controls were collected between August 2011 and August 2013. Swabs were tested for respiratory pathogens, including HRV, using quantitative real-time PCR assays. HRV-positive samples were sequenced for phylogenetic analysis by targeting the 5′ noncoding region (5′NCR). Our data showed that there were no differences in the prevalence of HRV detection among cases and controls (21% versus 20%, *P = *0.693); however, among children 13 to 59 months old, HRV detection was more often case associated (21% versus 16%; *P = *0.009), with the results mainly driven by HRV-C (12% versus 7%; *P = *0.001). Overall, there were no differences in the results of molecular subtyping of the HRV species prevalence among cases (for HRV-A, 48%; for HRV-B, 7%; for HRV-C, 45%) and controls (for HRV-A, 45%; for HRV-B, 10%; for HRV-C, 45% [*P = *0.496]). Those with pneumonia and HRV-C were older (12.1 versus 9.4 months, *P = *0.033) and more likely to present with wheeze (35% versus 25%, *P = *0.031) than those with HRV-A cases. Thus, the rate of HRV detection was high, with similar degrees of genetic diversity among cases and controls, confounding the interpretation of the presence of HRV in nasopharyngeal samples for attribution of a causal role in the pathogenesis of severe pneumonia in infants. However, among children 13 to 59 months of age, HRV detection, in particular, HRV-C detection, was associated with case status, especially among children with wheezing disease.

## Abstract (precis)

The pathogenesis of human rhinovirus (HRV) during severe respiratory disease remains undefined; thus, we aimed to explore the relationship between the HRV molecular subtyping results obtained during severe and asymptomatic childhood infections. Nasopharyngeal/oropharyngeal swabs from children (1 to 59 months of age) hospitalized with pneumonia and from age-frequency-matched controls were collected between August 2011 and August 2013.

## INTRODUCTION

Pneumonia is the leading cause of childhood morbidity and mortality globally, including approximately 120 million cases annually (1). Determining the etiology of pneumonia is difficult; traditionally, no causative pathogens have been identified in 20% to 60% of children with pneumonia (2–4). Nevertheless, respiratory viruses are associated with 14% to 80% of childhood community-acquired pneumonia episodes in younger children (2), with human rhinovirus (HRV) being detected most commonly.

HRV was first identified in 1956 in patients presenting with mild upper respiratory tract infection (URTI) (5) and has since been reported to be the most widespread cause of the common cold in children and adults. HRV belongs to the *Picornaviridae* family in the *Enterovirus* genus and is made up of three species, namely, HRV-A, HRV-B, and HRV-C (6–9). There are currently over 100 known HRV serotypes classified into the three species based on their nucleotide sequence homologies; this considerable serotype diversity could account for the differences in clinical presentation of HRV infection (10).

In this study, we aimed to compare results of molecular subtyping of HRV in children 1 to 59 months of age with severe/very-severe pneumonia and age-frequency-matched community controls enrolled in the parent Pneumonia Etiology for Child Health (PERCH) study.

## MATERIALS AND METHODS

### Case and control definitions.

The cases were children (1 to 59 months of age) hospitalized with severe or very-severe pneumonia based on World Health Organization definitions (11, 12), and the controls were children who were enrolled from the same community and were HIV status and age group and frequency matched to the cases. The control population was broken up into two groups: the URTI controls, i.e., those who had signs or symptoms of cough, runny nose, ear discharge, or wheezing or difficulty breathing together with either a fever (temperature, ≥38°C) or a sore throat reported in the past 48 h, and the non-URTI controls, i.e., those who appeared healthy for 48 h prior to sampling.

Comprehensive details on case and control enrollment criteria, sample testing, and clinical evaluation in the PERCH study have been described previously (13, 14). This study was limited to children enrolled in three sub-Saharan PERCH African countries, i.e., Mali, South Africa, and Zambia.

### Specimen collection and laboratory testing.

All cases and controls were sampled for blood and with a flocked nasopharyngeal (NP) swab (flexible minitip) (Copan catalogue no. 516 C) together with a rayon oropharyngeal (OP) swab (Fisher Scientific) on enrollment into the study. The NP sample was collected by swabbing the posterior nasopharynx, and the OP sample was collected by swabbing the posterior oropharynx and the tonsillar pillars. Both swabs were placed in the same 3 ml of universal transport media (Copan) and processed within 24 h of collection. Samples were stored at 4°C until processing and then archived at −70°C until testing and for long-term storage. A NucliSens EasyMag extraction system was used to extract the total nucleic acids from 400 μl of the NP/OP swab samples using the generic on-board extraction protocol and from 200 μl of blood samples using the specific-B extraction protocol, and the nucleic acids were eluted into a final volume of 110 μl (bioMérieux, Marcy l'Etoile, France). NP/OP samples were tested in each country using an FTD Resp-33 real-time quantitative PCR kit supplied by Fast-Track Diagnostics (Sliema, Malta) per the instructions of the manufacturer (15), and blood samples were tested for the presence of Staphylococcus pneumoniae as previously described (16). Standard curves were used to calculate the pathogen load using cycle threshold PCR quantifications as previously described (17), and all results were log_10_ transformed. Full blood counts and C-reactive protein (CRP) and blood culture testing using a BacT/Alert microbial system (bioMérieux, Marcy l'Etoile, France) were conducted for all cases. Where clinically indicated, pleural fluids, gastric aspirates, and lung aspirates were collected and cultured using standard culture and biochemical tests. The pleural fluids and lung aspirates were also tested using FTD-33 respiratory panels and the BinaxNow antigen assay per the instructions of the manufacturer (Alere, Orlando, FL).

Microbiologically confirmed pneumococcal pneumonia (MCPP) was defined as detection of S. pneumoniae cultured from a normally sterile fluid. In addition, a case was considered to have MCPP if the pleural fluid or lung aspirate was positive for pneumococcus with the BinaxNow or the FTD-33 assay. In another PERCH analysis, it was determined that there is an association between high pneumococcal densities in the nasopharynx or the whole-blood (WB) samples and the presence of MCPP in the pneumonia cases (18). It was determined that the optimal nasopharynx colonization and WB density thresholds for differentiating between MCPP cases and controls were >6.9 log_10_ copies/ml and >2.2 log_10_ copies/ml, respectively. These high-density pneumococcal (HDP) thresholds were used as markers for likely MCPP in order to analyze the relationship between HRV and pneumococcus disease.

### Determination of HRV molecular subtyping.

HRV molecular subtyping was performed as previously described (19, 20) using primers (DK001 forward and DK004 reverse) targeting the 5′ noncoding region (5′NCR) of the HRV region for PCR amplification and subsequent sequencing. The ClustalW algorithm implemented in Geneious 4.7.6 (21) was used to identify the HRV species, and MEGA-6 (22) was used to construct the phylogenetic trees using Kimura’s 2-parameter technique with bootstrap values estimated using 1,000 bootstrap replications (23).

### Statistical analysis.

Demographic characteristics of cases and controls were analyzed using chi-square and Wilcoxon tests, and the levels of prevalence of the HRV species were quantified using binary multinomial regression and odds ratio determinations. Continuous variables were tested using *t* tests and multivariate regression models. Univariate analysis was performed prior to multivariate analysis, and all characteristics with an association corresponding to a *P* value of <0.2 were included. STATA version 12 (College Station, TX, USA) was used for all statistical analysis, and a two-sided *P* value of <0.05 was considered statistically significant.

### Data availability.

The sequences for all rhinovirus-positive samples have been deposited in GenBank with accession numbers MK858576 to MK858936 and MK858937 to MK859375.

## RESULTS

### The study population.

A total of 2,120 hospitalized cases with severe/very-severe pneumonia were enrolled across the three sub-Saharan African sites included in this study. Of these children, 439 (21%) tested positive for HRV and were subtyped. The prevalence of HRV-associated pneumonia was highest in South Africa (23%, *n* = 210/911), followed by Zambia (21%, *n* = 113/538), and was lowest in Mali (17%, *n* = 116/671, *P = *0.020) among the pneumonia cases. Additionally, 2,284 age-frequency-matched community controls were enrolled across the sites, among whom the levels of prevalence of HRV infection (overall, 20%, *n* = 462) were similar across the sites, including 22% in South Africa (*n* = 212/964), 20% in Mali (*n* = 143/725), and 18% in Zambia (*n* = 107/686, *P = *0.093).

There were no differences in the prevalences of HRV detection and HRV load between cases (21% and 3.6 log_10_ copies/ml, respectively) and controls (20% and 3.5 log_10_ copies/ml; *P = *0.334 and *P = *0.08, respectively). Furthermore, the prevalence of HRV identification, the species distribution, and the viral load did not differ between cases and controls at the individual sites (data not shown). However, among children >12 months of age, the cases had a significantly greater prevalence of HRV detection (21%) than the controls (16%, *P = *0.009), with the results driven by HRV-C detection (12% versus 7%, *P = *0.001) (Table 1).

**TABLE 1:** Molecular subtyping of the HRV population in cases and community controls stratified by age groups^*a*^

Category	Total no. of subjects	No. (%) of subjects with HRV detected or *P* value				*P* value^*b*^
		Overall (*n* = 901)	HRV-A (*n* = 389)	HRV-B (*n* = 71)	HRV-C (*n* = 376)	
All ages						
Cases	2,120	439 (21)	199 (9)	31 (1)	185 (8)	0.496
Controls	2,284	462 (20)	190 (8)	40 (2)	191 (8)	
*P* value^*c*^		0.693	0.425	0.306	0.861	

1–5 mo						
Cases	1,050	207 (20)	110 (10)	21 (2)	67 (6)	0.270
Controls	879	201 (23)	88 (10)	24 (3)	74 (8)	
*P* value^*c*^		0.223	0.107	0.474	0.246	

6–12 mo						
Cases	507	112 (22)	46 (9)	8 (2)	51 (10)	0.921
Controls	608	135 (22)	54 (9)	8 (1)	63 (10)	
*P* value^*c*^		0.964	0.925	0.650	0.750	

13–59 mo						
Cases	563	120 (21)	43 (7)	2 (0)	67 (12)	0.072
Controls	797	126 (16)	48 (6)	8 (1)	54 (7)	
*P* value^*c*^		0.009	0.227	0.181	0.001	

a *P* values were determined by chi-square tests. Logistic regression models were adjusted for confounding variables (*P* values < 0.2 in univariate analysis). HRV, human rhinovirus.

b Data represent *P* values for differences between cases and controls across the three HRV species.

c Data represent *P* values for comparisons of individual HRV species between cases and controls for the different age groups.

**FIG 1:**
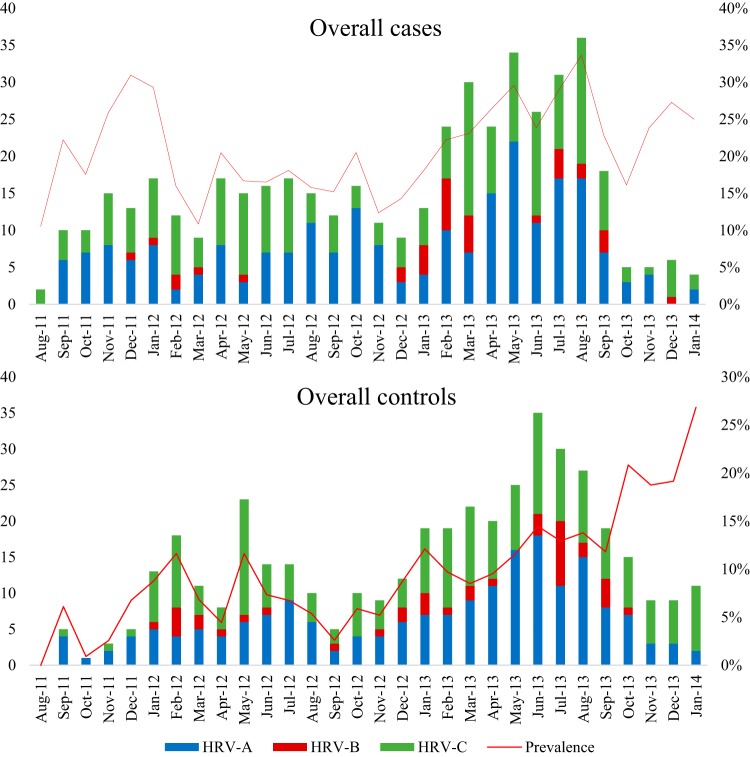
Seasonal distribution of HRV species over the study period in children hospitalized with pneumonia and age-matched community controls. Prevalence data represent numbers of HRV-positive participants in relation to the total number of tested samples for each month.

The seasonal circulation of the three HRV species over the full study period for cases and controls is detailed in Fig. 1. For both cases and controls, HRV-B appeared sporadically throughout the year with no obvious seasonality pattern. Similarly, there were no obvious patterns in the circulation of the HRV-A and HRV-C species among both cases and controls; however, they were detected throughout the year. Furthermore, no obvious pattern or clear seasonal distribution of HRV infection was observed at any of the sites (see Fig. S1 in the supplemental material). The overall prevalence for HRV ranged from 11% in August 2011 and March 2012 to 33% in August 2013 among cases and from 1% in October 2011 to 27% in January 2014 among controls during the study period.

### HRV clinical and molecular subtyping among community controls.

Among the HRV-positive samples from community controls, 97% (*n* = 446) were successfully amplified, 3% (*n* = 14) failed to amplify, and 0.5% (*n* = 2) were of insufficient volume for serotyping. Among the amplified samples, 5% were typed as enterovirus and one as echovirus and 92% as HRV. The dominant HRV species among controls were HRV-C (45%) and HRV-A (45%), and 10% were HRV-B. There were no differences in species distribution between asymptomatic controls (HRV-A, 47%; HRV-B, 10%; HRV-C, 43%) and controls with signs and symptoms of upper respiratory tract infections (including runny nose, cough, wheeze, difficulty breathing, and fever) (HRV-A, 38%; HRV-B, 6%; HRV-C, 56%; *P = *0.066).

There were no differences in demographic and clinical symptom characteristics among controls with HRV-B infection compared to those with HRV-A and HRV-C infection. Comparisons of controls to those with HRV-A and HRV-C infections showed that, other than for controls with HRV-A being more likely to be *LytA* positive on whole blood (10% versus 4%, *P = *0.012), there were no differences in demographic characteristics, HRV load, and symptoms of RTI (Table 2).

**TABLE 2:** Demographic and clinical characteristics of the community controls infected with the three HRV species^*a*^

Characteristic	Value(s)			Unadjusted *P* value	aOR (95% CI)	Adjusted *P* value
	HRV-A (*n* = 190)	HRV-B (*n* = 40)	HRV-C (*n* = 191)			
Age in mo, mean (SD)	9.7 (9.9)	7.3 (7.6)	10.8 (10.7)	0.300		0.230
No. (%) female	93 (49)	23 (58)	89 (47)	0.646	1.04 (0.69–1.57)	0.854
No. (%) HIV^+^	14 (7)	1 (3)	13 (7)	0.967	0.99 (0.47–2.24)	0.999
No. (%) HEU^*b*^	32 (18)	6 (15)	39 (22)	0.382	0.60 (0.34–1.26)	0.178
No. (%) never breast fed	49 (26)	12 (30)	49 (26)	0.340	0.82 (0.50–1.34)	0.420
No. (%) underweight^*c*^	22 (12)	7 (18)	16 (8)	0.299	1.67 (0.83–3.36)	0.151
No. (%) attending day care	44 (23)	14 (35)	65 (34)	0.023	0.74 (0.40–1.35)	0.323
No. (%) with smoker in household	53 (28)	6 (15)	52 (27)	0.884	0.97 (0.62–1.54)	0.905
No. (%) with premature birth^*d*^	32 (17)	6 (15)	34 (18)	0.795	0.95 (0.79–1.15)	0.618
Birth wt (kg), mean (SD)	2.9 (0.6)	3.0 (0.7)	3.1 (0.5)	0.196		0.328
No. (%) with clinical feature:						
Tachypnea^*e*^	16 (9)	5 (13)	13 (7)	0.530	1.37 (0.62–3.0)	0.433
Cough	13 (7)	2 (5)	13 (7)	0.993	1.14 (0.50–2.57)	0.757
Fever^*f*^	1 (1)	0	3 (2)	0.343	0.44 (0.04–4.39)	0.481
Diarrhea	1 (1)	1 (3)	0	0.364		0.462
Rhinorrhea	18 (9)	4 (10)	33 (17)	0.605	0.69 (0.34–1.41)	0.310
Asymptomatic^*g*^	29 (37)	5 (6)	45 (57)	0.042	1.25 (0.69–2.27)	0.464
No. (%) with laboratory marker:						
CRP ≥ 40mg/l^*h*^	2 (1)	0	2 (1)	0.996	1.22 (0.16–9.0)	0.847
*LytA* positive^*i*^	19 (10)	4 (10)	7 (4)	0.019	3.25 (1.30–8.14)	0.012
No. (%) with HDP^*j*^						
Blood	12 (6)	3 (8)	5 (3)	0.092	2.88 (0.97–8.16)	0.058
NP	29 (15)	3 (8)	35 (18)	0.425	0.81 (0.47–1.41)	0.458
HRV load, mean (SD)^*k*^	3.5 (0.9)	3.3 (0.7)	3.5 (0.9)	0.542		0.138
HRV monoinfection^*l*^	130 (68)	27 (67)	123 (64)	0.406	1.10 (0.70–1.68)	0.713
				
No. (%) with viral coinfection in the nasopharynx with:						
AdV	17 (9)	0	22 (12)	0.409	0.85 (0.43–1.68)	0.635
RSV	5 (3)	4 (10)	5 (3)	0.993	0.93 (0.26–3.31)	0.915
HBoV	21 (11)	2 (5)	25 (13)	0.542	0.92 (0.49–1.76)	0.797
HMPV	4 (2)	3 (8)	0	0.044		0.168
InFV A–C	1 (1)	2 (5)	0	0.179		0.175
PIVs	4 (2)	3 (8)	12 (6)	0.050	3.81 (1.17–12.42)	0.026
HCoVs	16 (8)	0	29 (15)	0.044	0.57 (0.29–1.10)	0.091

No. (%) with bacterial coinfection in the nasopharynx with:						
Staphylococcus pneumoniae	150 (79)	27 (68)	156 (82)	0.503	0.97 (0.57–1.63)	0.887
Staphylococcus aureus	30 (16)	12 (30)	21 (11)	0.171	1.40 (0.75–2.58)	0.290
Moraxella catarrhalis	139 (73)	28 (70)	153 (80)	0.110	0.78 (0.47–1.30)	0.336
Haemophilus influenzae	104 (55)	16 (40)	96 (50)	0.382	1.39 (0.91–2.12)	0.131

a Abbreviations: HIV, human immunodeficiency virus; HEU, HIV uninfected but HIV exposed; OR, odds ratio; aOR, adjusted odds ratio; CI, confidence interval; SD, standard deviation; HDP, high-density pneumococcus; CRP, C-reactive protein; NP, nasopharyngeal; HRV, human rhinovirus; RSV, respiratory syncytial virus (A and B), HMPV, human metapneumovirus; AdV, adenovirus; PIVs, parainfluenza virus types 1 to 4; HBoV, human bocavirus; HCoV, human coronavirus (OC43, NL63, 229E, and HKU1); InFV A–C, influenza virus A, B, and C. *P* values were calculated by comparing HRV-A-infected subjects to HRV-C-infected subjects using chi-square and Wilcoxon tests with logistic regression models adjusted for confounding variates (*P* values < 0.2 in univariate analysis) where applicable. Odds ratios could not be calculated for variables with zero values.

b HIV uninfected but HIV exposed (HEU) was defined as a negative virologic test result for HIV in a patient who had evidence of HIV exposure, defined as HIV seropositivity (if <12 months of age) or seronegativity with a maternal history of HIV infection (for all ages), with the caveat that maternal exposure must be confirmed by maternal serology for seronegative infants aged <7 months.

c Underweight was defined as weight for age ≤2 SD of the median age-sex-specific WHO reference.

d Premature birth was defined as gestational age <37 weeks.

e Tachypnea was defined as respiratory rate >60 breaths/min for subjects aged <2 months, respiratory rate >50 breaths/min for subjects aged 2 to 12 months, and respiratory rate >40 breaths/min for subjects aged >12 months.

f Fever was defined as temperature ≥38°C.

g The child was completely asymptomatic for all signs of respiratory tract illness, including runny nose, fever, cough, wheezing, and difficulty breathing.

h CRP was defined as levels ≥40 mg/liter, which are considered to show potential bacterial infection. Only a subset of randomly chosen controls had CRP testing conducted at the South African site.

i Blood sample positive for S. pneumoniae colonization by *LytA* PCR.

j HDP was defined as S. pneumoniae density in nasopharynx >6.9 log_10_ copies/ml and/or density in whole-blood sample >2.2 log_10_ copies/ml.

k HRV load in the nasopharynx, expressed as log_10_ copies/ml.

l HRV was the only respiratory virus detected in the nasopharynx.

HRV was the sole respiratory virus identified in the nasopharynx in 67% of the controls with HRV infection, including 68%, 67%, and 64% with HRV-A, HRV-B, and HRV-C infections, respectively (*P = *0.713) (Table 2). However, among respiratory virus coinfections, coinfection with parainfluenza virus (PIV) was more common among controls with HRV-C (6%) than among those with HRV-A (2%, *P = *0.026) (Table 2).

Further, when the HRV types were compared among the asymptomatic and RTI controls separately, there were no significant differences for any of the demographic, clinical, or molecular markers (Table S1 and S2).

### Molecular subtyping of the HRV-associated cases.

Among the HRV-associated pneumonia cases, 97% of the samples were successfully subtyped, while 3% (*n* = 11) of the samples failed to amplify, all of which had very low copy numbers (threshold cycle [*C_T_*] value, >37) in the real-time PCR assay. Of the 428 amplified samples, 3% (*n* = 13) were typed as enterovirus and closely related member of HRV. The species of samples was successfully identified in 91% (*n* = 415) of the HRV-associated cases, among which 48%, 45%, and 7% were found to be infected with HRV-A, HRV-C, and HRV-B (7%), respectively (Table 1). The distribution of HRV species differed by age group, with HRV-A (52%) being the most prevalent among infants <12 months of age, followed by HRV-C (38%) and then HRV-B (10%), whereas HRV-C (60%) was more prevalent among children 13 to 59 months of age, followed by HRV-A (38%) and HRV-B (2%, *P = *0.002).

Similarly to the results seen with the controls, HRV-B was the least prevalent species and appeared sporadically throughout the 2-year period (Fig. 1). This limited any in-depth statistical analysis specific to HRV-B, with no significant differences in demographic and clinical characteristics observed between HRV-B-associated cases and HRV-A-associated or HRV-C-associated cases, except that the subjects with HRV-B infection were younger (4.8 months of age) than the subjects with HRV-A infection (9.4 months, *P = *0.01) and with HRV-C infection (12.1 months, *P < *0.001) (Table 3).

**TABLE 3:** Demographic and clinical characteristics of subjects infected with HRV-A, HRV-B, and HRV-C^*a*^

Characteristic	Value(s)			Unadjusted *P* value	aOR (95% CI)	Adjusted *P* value
	HRV-A (*n* = 199)	HRV-B (*n* = 31)	HRV-C (*n* = 185)			
Age in mo, mean (SD)	9.4 (9.5)	4.8 (4.9)	12.1 (10.2)	0.023		0.033
No. (%) female	82 (41)	17 (55)	92 (50)	0.094	0.72 (0.48–1.10)	0.126
No. (%) HIV positive	30 (15)	2 (6)	16 (9)	0.044	1.96 (0.98–3.94)	0.059
No. (%) HEU^*b*^	41 (24)	3 (10)	45 (27)	0.617	0.93 (0.53–1.62)	0.786
No. (%) never breast fed	40 (20)	4 (13)	27 (14)	0.157	1.78 (1.02–3.11)	0.099
No. (%) underweight^*c*^	71 (36)	7 (23)	51 (28)	0.089	1.54 (0.98–2.42)	0.061
No. (%) attending day care	55 (28)	7 (23)	43 (23)	0.331	1.63 (0.87–3.05)	0.126
No. (%) with smoker in household	69 (35)	9 (29)	56 (30)	0.421	1.28 (0.81–2.0)	0.290
No. (%) with premature birth^*d*^	26 (13)	2 (6)	15 (8)	0.248	1.43 (0.76–2.71)	0.266
Birth wt (kg), mean (SD)	2.9 (0.7)	3.0 (0.7)	3.0 (0.6)	0.237		0.235

No. (%) with clinical feature:						
Very severe pneumonia	88 (44)	12 (39)	72 (39)	0.293	1.31 (0.85–2.01)	0.265
CXR abnormal^*e*^	91 (46)	15 (48)	66 (36)	0.046	1.57 (1.02–2.41)	0.040
Supplementary 0_2_ therapy	113 (57)	20 (65)	110 (59)	0.596	0.92 (0.49–1.70)	0.791
Mechanical ventilation	6 (3)	0	7 (4)	0.678	0.58 (0.18–1.86)	0.362
Hypoxia^*f*^	108 (55)	23 (74)	120 (60)	0.302	0.76 (0.49–1.19)	0.206
Tachycardia^*g*^	113 (57)	17 (55)	108 (58)	0.796	0.95 (0.62–1.46)	0.818
Tachypnea^*h*^	169 (86)	24 (77)	161 (87)	0.818	1.09 (0.59–2.01)	0.931
Wheezing	49 (25)	7 (23)	65 (35)	0.025	0.61 (0.39–0.95)	0.031
Cough	142 (71)	23 (74)	139 (75)	0.339	0.82 (0.50–1.34)	0.494
Lethargy	26 (13)	3 (10)	15 (8)	0.119	1.97 (0.98–3.96)	0.056
Fever^*i*^	152 (76)	23 (74)	139 (75)	0.776	1.04 (0.64–1.69)	0.901
Convulsions	14 (7)	2 (6)	4 (2)	0.033	2.78 (0.82–9.45)	0.098
Diarrhea	51 (26)	8 (26)	26 (14)	0.005	2.23 (1.24–4.02)	0.007
Head nodding	54 (27)	9 (29)	51 (28)	0.924	1.00 (0.63–1.61)	0.947
Central cyanosis	9 (5)	1 (3)	3 (2)	0.118	2.50 (0.64–9.62)	0.199
Inability to feed	17 (9)	1 (3)	17 (9)	0.824	0.87 (0.41–1.86)	0.611
Vomiting everything	4 (2)	0	2 (1)	0.470	2.17 (0.37–12.55)	0.510
Lower chest wall indrawing	183 (92)	28 (90)	176 (95)	0.212	0.46 (0.19–1.10)	0.078
Stridor	7 (4)	1 (3)	6 (3)	0.684	0.76 (0.26–2.22)	0.875
Grunting	46 (23)	10 (32)	46 (25)	0.597	0.75 (0.43–1.30)	0.333
Nasal flaring	152 (76)	24 (77)	148 (80)	0.392	0.83 (0.50–1.40)	0.575

Laboratory marker:						
Leucocytosis^*j*^	88 (44)	13 (42)	75 (41)	0.524	1.27 (0.82–1.97)	0.209
CRP ≥ 40 mg/liter^*k*^	58 (29)	10 (32)	47 (25)	0.412	1.32 (0.83–2.10)	0.267
Blood culture positive^*l*^	10 (5)	5 (16)	11 (6)	0.692	0.83 (0.33–2.04)	0.623
*LytA* positive^*m*^	19 (9)	4 (12)	15 (7)	0.514	1.30 (0.60–2.82)	0.469
MCPP^*n*^	6 (3)	0	2 (1)	0.204	3.78 (0.70–20.50)	0.143
HDP^*o*^						
Blood	13 (7)	3 (10)	9 (5)	0.433	1.40 (0.57–3.43)	0.471
NP	38 (19)	4 (13)	24 (13)	0.105	1.72 (0.96–3.09)	0.069
No. (%) with hospital stay >3 days	141 (71)	23 (74)	117 (63)	0.113	1.34 (0.85–2.10)	0.234
Case fatality ratio	32 (21)	2 (9)	25 (16)	0.332	1.23 (0.66–2.29)	0.522
HRV load, mean (IQR)^*p*^	3.5 (3.0–4.1)	3.5 (2.8–3.8)	3.5 (3.1–4.3)	0.544		0.846
No. (%) with HRV monoinfection	110 (55)	13 (42)	100 (54)	0.810	1.07 (0.70–1.63)	0.742
				
No. (%) with viral coinfection in the nasopharynx with:						
AdV	17 (9)	3 (10)	30 (16)	0.024	0.58 (0.29–1.14)	0.116
RSV	36 (18)	8 (26)	24 (13)	0.169	1.14 (0.62–2.07)	0.679
HBoV	18 (9)	5 (16)	31 (17)	0.029	0.58 (0.30–1.10)	0.095
HMPV	5 (3)	1 (3)	6 (3)	0.669	0.71 (0.20–2.45)	0.592
InFV A–C	2 (1)	0	2 (1)	0.942	1.06 (0.14–7.98)	0.954
PIVs	18 (9)	2 (6)	6 (3)	0.024	2.82 (1.07–7.40)	0.036
HCoV	11 (6)	1 (3)	14 (8)	0.420	0.70 (0.30–1.63)	0.414
No. (%) with bacterial coinfection in the nasopharynx with:						
Staphylococcus pneumoniae	145 (73)	19 (61)	138 (75)	0.700	0.97 (0.58–1.62)	0.903
Staphylococcus aureus	199 (40)	6 (19)	37 (20)	0.980	0.66 (0.36–1.19)	0.168
Moraxella catarrhalis	139 (70)	19 (61)	118 (64)	0.207	1.38 (0.85–2.25)	0.197
Haemophilus influenzae	109 (55)	14 (45)	95 (51)	0.502	1.17 (0.77–1.78)	0.453

a Abbreviations: HRV, human rhinovirus; OR, odds ratio; aOR, adjusted odds ratio; CI, confidence interval, SD, standard deviation; IQR, interquartile range; HIV, human immunodeficiency virus; HEU, HIV uninfected but HIV exposed; CXR, chest X-ray; CRP, C-reactive protein; MCPP, microbiologically confirmed pneumococcal pneumonia; HDP, high-density pneumococcus; NP, nasopharyngeal; RSV, respiratory syncytial virus, HMPV, human metapneumovirus; AdV, adenovirus; PIVs, parainfluenza virus types 1 to 4; HBoV, human bocavirus; HCoV, human coronavirus (OC43, NL63, 229E, and HKU1); InFV A–C, influenza virus A, B, and C. Odds ratios and *P* values were calculated by comparing HRV-A-infected subjects to HRV-C-infected subjects using chi-square and Wilcoxon tests. Logistic regression models were adjusted for confounding variates (<0.2 in univariate analysis) where applicable. Odds ratio could not be calculated for continuous variables or variables with 0 values; thus, the corresponding cells were left blank.

b HIV uninfected but HIV exposed (HEU) was defined as a negative virologic test result for HIV in a patient who had evidence of HIV exposure, defined as HIV seropositivity (if <12 months of age) or seronegativity with a maternal history of HIV infection (for all ages), with the caveat that maternal exposure must be confirmed by maternal serology for seronegative infants aged <7 months.

c Underweight was defined as weight for age ≤2 SD of the median age-sex-specific WHO reference.

d Premature birth was defined as gestational age <37 weeks.

e Abnormal chest X-ray was defined as radiographically confirmed endpoint pneumonia consolidation or presence of any infiltrates.

f Hypoxia was defined as (i) a room air pulse-oximetry reading indicating oxygen saturation at <90% at the two sites at elevation (Zambia and South Africa) or at <92% at all other sites or (ii) absence of a room air oxygen saturation reading and child on oxygen.

g Tachycardia was defined as heart rate >160 beats per min (bpm) for subjects aged <11 months, heart rate >150 bpm for subjects aged 12 to 35 months, or heart rate >140 bpm for subjects aged 36 to 59 months.

h Tachypnea defined as respiratory rate >60 breaths/min for subjects aged <2 months, respiratory rate >50 breaths/min for subjects aged 2 to 12 months, or respiration rate >40 breaths/min for subjects aged >12 months.

i Fever was defined as temperature >38°C.

j Leucocytosis was defined as white blood cell count >15,000 cells/μl for subjects aged <12 months or white blood cell count >13,000 cells/μl for subjects aged >12 months.

k CRP was defined as levels ≥40 mg/ml, which are considered to potentially indicate bacterial infection.

l Blood culture positive for any significant noncontaminate bacteria.

m Blood sample positive for S. pneumoniae colonization by *LytA* PCR.

n MCPP, S. pneumoniae cultured from a normally sterile body fluid—blood, pleural fluid, or lung aspirate—or pleural fluid or lung aspirate gave a PCR *LytA*-positive result.

o HDP defined as S. pneumoniae density in nasopharynx >6.9 and/or density in whole-blood sample >2.2 log10 copies/ml.

p HRV load in the nasopharynx (expressed as log_10_ copies per milliliter).

Further species-specific analyses were limited to comparing HRV-A-associated cases to HRV-C-associated cases, with HRV-A cases being younger (9.4 months) than HRV-C cases (12.1 months, *P = *0.033) cases (Table 3). Furthermore, the HRV-A-associated cases were more likely to have radiographically confirmed pneumonia (abnormal chest radiograph, defined as primary endpoint pneumonia or presence of any infiltrates) than the HRV-C-associated cases (46% versus 36%, *P = *0.040) and were more likely to present with concurrent diarrhea (26% versus 14%, *P = *0.007). In contrast, cases with HRV-C were more likely to present with wheeze (35%) than cases with HRV-A (25%, *P = *0.031) (Table 3). Among the HRV-associated cases, HRV was the only virus identified in the nasopharynx samples from 54% (*n* = 223/415) of the cases, including 55%, 42%, and 54% of HRV-A, HRV-B, and HRV-C infections, respectively (*P = *0.742).

### Case-control comparison of results of molecular subtyping of HRV.

**FIG 2:**
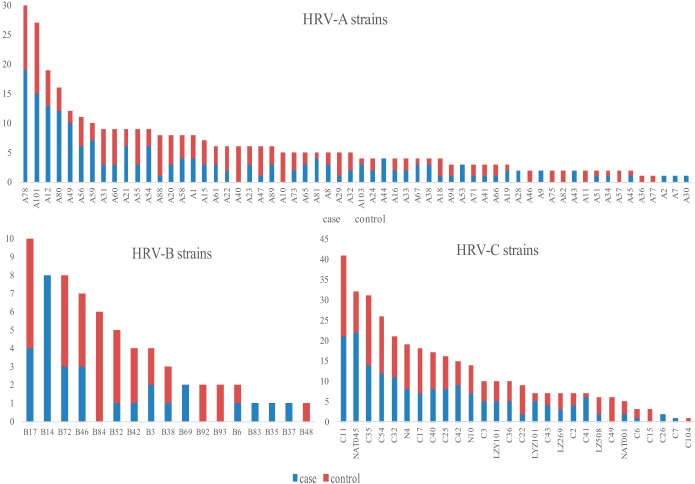
Frequency of HRV strains. The prevalence of detected types in cases and controls is indicated for each species. Prevalence data represent numbers of HRV-positive participants in relation to the total number of tested samples for each month.

There were 60 different HRV-A strains, 17 different HRV-B strains, and 28 different HRV-C strains circulating throughout the 2-year period (Fig. 2). No discernible differences were noted in the distribution of strains between cases and controls; moreover, there were no apparent differences in the distribution of strains among the three sites (Fig. S2 to S4). Additionally, no obvious patterns of temporal clustering of HRV species or strains were observed, with strain distributions differing on a month-to-month basis (data not shown).

The HRV sequences for each of the species formed many unique clusters, with mean levels of nucleotide diversity of 82% for HRV-A (nucleotide diversity range, 45% to 100%), 80% for HRV-B (nucleotide diversity range, 53% to 100%), and 74% for HRV-C (nucleotide diversity range, 52% to 100%). The levels of nucleotide diversity did not differ among cases and controls for the different HRV species. The HRV-A and HRV-B sequences clustered with the GenBank sequences of known HRV-A and HRV-B strains, with statistically significant bootstrap support, whereas the HRV-C sequences tended to form numerous subclusters which did not always cluster closely with the GenBank sequences but which did always occur in the same monophyletic groups of known HRV-C strains. The numerous clusters and ranges of nucleotide diversities, especially in the HRV-C population, suggest considerable diversity in the strains present in the population (Fig. S2 to S4).

Among the HRV-A strains, the nucleotide similarities to the closest GenBank prototype reference strains ranged from 82.3% to 99.4% and strain identities with other contemporaneous HRV-A strains ranged from 79.8% to 100%. Among the HRV-B strains, the nucleotide similarities to the closest GenBank prototype reference strains ranged from 87.9% to 99.8% and strain identity with other contemporaneous HRV-B strains ranged from 91.2% to 100%. Among the HRV-C strains, the nucleotide similarities to the closest GenBank prototype reference strains showed much lower levels of relatedness than the other two species and ranged from 74.5% to 98.8%; however, there were high degrees of similarity with the other contemporaneous HRV-C strains, with strain identities ranging from 93.9% to 100%. No novel serotypes were identified in this study.

## DISCUSSION

This report presents results of genotyping of 836 HRV-positive samples, including 415 from children hospitalized with severe or very-severe pneumonia and 421 from community controls, representing the largest and most in-depth case-control study reporting on HRV molecular subtyping to date. HRV-A was the dominant species identified among cases (48%) and controls (45%), followed closely by HRV-C (45% each among cases and controls), whereas HRV-B was seen only intermittently and accounted for 7% and 10% of HRV strains among cases and controls, respectively.

Among the three sub-Saharan countries, the overall prevalences of HRV detection and viral loads did not differ between cases (21%) and controls (20%). However, presence of HRV in the nasopharynx was associated with case status (21% versus 16% among controls) among children in the age group of 12 to 59 months; among those children, a higher percentage of HRV were HRV-C species in cases (12%) than in controls (7%). Furthermore, HRV loads did not differ significantly between the HRV species, and none of the HRV species were more likely to be associated with either more bacterial coinfections or more viral coinfections regardless of case or control status. The HRV species distributions among cases and controls in our study in both hospitalized and control populations are similar to those reported previously by others (24–28). Notably, a number of the HRV-positive cases (8%) and controls (7%) were also positive for S. pneumoniae detection in the blood; those levels are comparable to the overall levels of S. pneumoniae detection in the whole of the PERCH study (7% and 6% for cases and controls, respectively) (16). Thus, the pneumococcal PCR had low specificity for diagnosing invasive pneumococcal disease in children, and more work is needed in order to understand the pathophysiology and implications of viral coinfections.

Previous smaller studies from Africa, Asia, Europe, America, and Australia (29–32) have suggested that HRV-C may cause more-severe illness and is more prevalent in cases of lower respiratory tract infections (LRTI) than HRV-A and HRV-B. This was not evident in our study, however, where both HRV-A and HRV-C were ubiquitous throughout the study period in cases and controls and were similarly prevalent among the cases. Additionally, there was no evidence that the cases associated with HRV-C infection had more-severe disease than the cases associated with HRV-A infection and with HRV-B infection, based on presence of hypoxia, presenting as very-severe pneumonia, prolonged hospital stay (>3 days duration), need for mechanical ventilatory support, or case fatality rate. Instead, among HRV-associated cases, those with HRV-A infection were more likely to have radiographically confirmed pneumonia and concurrent diarrhea than those with HRV-C infection.

That HRV-C infection is more common in older children hospitalized with pneumonia has also been reported by others (25, 33, 34). It has been suggested previously that the association between HRV-C and older children might be linked to asthma exacerbation and wheezing in these children. Previous studies have reported that HRV-C is more commonly associated with wheezing exacerbation than HRV-A or HRV-B (33–36). Similarly, in our study, the presence of wheezing was 1.64-fold more common among cases with HRV-C infection than among cases with HRV-A infection. This suggests that some of the children fulfilling our study-specified definition of “pneumonia” might instead have had reactive airway disease following HRV-C infection. This could have been the case despite our study having been designed to exclude likely asthma cases by performance of a bronchodilator nebulizer inhalation challenge prior to study enrollment; i.e., children in whom the lower chest wall indrawing resolved post-B2-agonist nebulization challenge, regardless of its effect on wheezing, were not enrolled.

These associations of HRV-A with more-severe LRTI and HRV-C with more wheezing disease have also been reported from a study in Burundi, where HRV-A was more prevalent among pneumonia and bronchitis cases and HRV-C among cases with acute wheezing (37). An association between HRV-C and wheezing disease was also seen in a study which characterized the cell receptors for HRV-C infection in humans, namely, the CDHR-3 receptors, which facilitate HRV-C adhesion and replication (16). A mutation (cysteine-to-tyrosine mutation at amino acid 529) in CDHR-3 enhances HRV-C binding and replication *in vivo* and has also been associated with increased susceptibility to wheezing and asthma illnesses (38).

In our study, HRV-A and HRV-C strains were present throughout the study period, with a highly heterogeneous population of over 100 strains identified. HRV-A, with 60 different strains, had the most diverse genetic population, followed by HRV-C with 28 strains and HRV-B with 17 different strains identified over the study period in both the cases and controls. Furthermore, there were no discernible relationships between the strains identified among cases and controls and no real evidence of temporal clustering of strains over time. However, this study was not sufficiently powered for statistical analysis of case-control status or clinical epidemiology among cases for the different strains making up each of the HRV species. Furthermore, we failed to type 3% (*n* = 25) of the HRV-positive samples (with similar percentages between cases and controls), which was largely due to PCR failure as a consequence of very low viral loads (*C_T_* >37). The possibility cannot be excluded that these PCR failures might have been due to variability in the primer annealing sites; thus, some genetic variants might have been missed by our typing assays. However, the typing failure rate of 3% is lower than that seen in other studies which looked at HRV serotyping by targeting the VP4-VP2 capsid region (15% to 44% failure) (19, 20, 26, 27). The 5′NCR serotyping technique has been found to have an increased sensitivity for HRV serotype detection in clinical samples compared to the more traditional VP4-VP2 PCR sequencing technique; further, it does not require a nested PCR or multiple primer pairs, thus reducing contamination rates, cost, and time required for serotyping (19).

Regardless, the considerable genetic diversity of HRV reported in this study highlights the heterogeneity of the HRV strains circulating within the general population and further emphasizes the difficulties in attributing causality of disease to HRV. The similarities in strains among cases and controls also negate the assertion that some strains might be more virulent. Other studies have also reported the diverse nature of the HRV genetic population among cases and controls, including studies from South Africa (28), Botswana (27), Kenya (26), and other countries (24, 25, 39) as well as the lack of obvious seasonal patterns among cases (35, 37, 40–42).

Study limitations included the lack of a gold standard for the determination of the actual cause of the pneumonia episode. Additionally, the cross-sectional design of the study means only a single specimen was taken upon admission to the hospital. The viral load is known to vary with time since onset of illness; thus, a longitudinal study would have allowed us to compare peak viral loads between subjects infected with the different HRV species and between cases with different levels of clinical severity. Furthermore, our study was not designed to analyze the role of HRV in the lower respiratory tract. Sampling the upper respiratory tract is more convenient and less invasive for the patient; however, detection of a pathogen and its viral load in these samples might not reflect the viral burden in the lung parenchyma. Direct sampling of the site of infection, namely, the lung, through lung aspirate sampling or bronchoalveolar lavage would provide more-direct evidence of the role of HRV in pneumonia; however, these sampling techniques are more invasive and difficult to perform in infants and young children. Animal models are needed to further study the pathogenicity of HRV infections.

In conclusion, this report emphasizes that the HRV populations circulating both in children hospitalized with pneumonia and in community controls are highly divergent, that similar strains are circulating over a large geographical location, and that the strains are similar year to year. Additionally, it further highlights the difficulty in attributing causality of LRTI disease to HRV in infants, as no differences were observed in the levels of prevalence of HRV detection between cases and controls (other than in the >12-month age group) and no differences were found in HRV genotypes between cases and controls, especially among infants. Nevertheless, among the cases, HRV-A tended to be more prevalent among younger children and was associated with more-severe disease and radiographically confirmed pneumonia compared to HRV-C infections, whereas HRV-C was more prevalent among older children with wheezing disease.

## Supplementary Material

Supplemental file 1

Supplemental file 2

Supplemental file 3

Supplemental file 4

Supplemental file 5

Supplemental file 6
