# Supplementary material for: Molecular Subtyping of Human Rhinovirus in Children from Three Sub-Saharan African Countries
Source: J Clin Microbiol. 2019 Aug 26;57(9):e00723-19. doi: 10.1128/JCM.00723-19 (PMC6711929; doi:10.1128/JCM.00723-19)
Supplement: Supplemental file 1 [file JCM.00723-19-s0001.pdf]

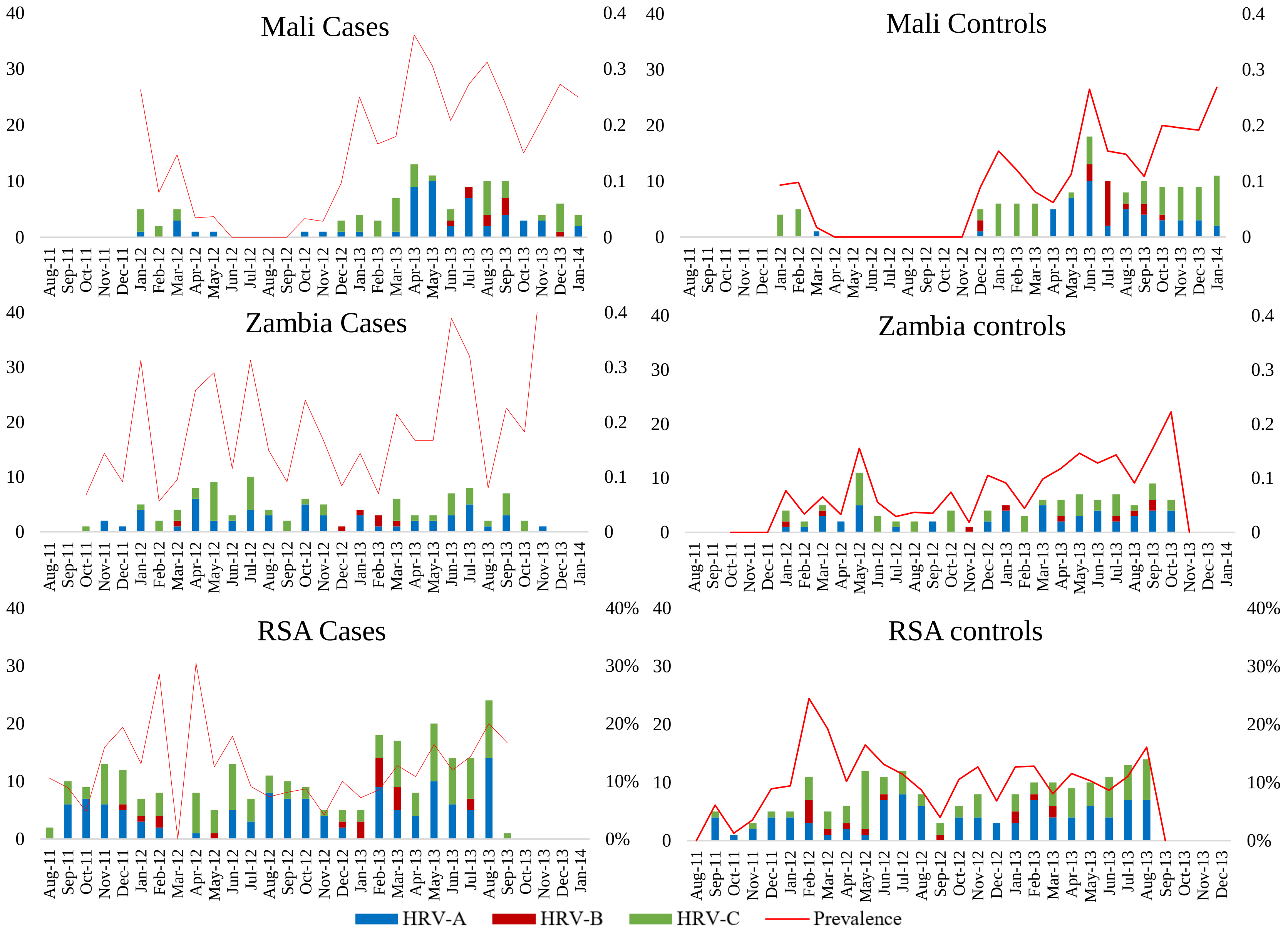

Supplementary Figure 1: The seasonal distribution of HRV species over the study period in Mali, Zambian and South African children hospitalised with pneumonia and age matched community controls.

Prevalence is the number of HRV positive participants in relation to the total number of tested samples for each month.
