# Supplementary material for: Molecular Subtyping of Human Rhinovirus in Children from Three Sub-Saharan African Countries
Source: J Clin Microbiol. 2019 Aug 26;57(9):e00723-19. doi: 10.1128/JCM.00723-19 (PMC6711929; doi:10.1128/JCM.00723-19)
Supplement: Supplemental file 2 [file JCM.00723-19-s0002.pdf]

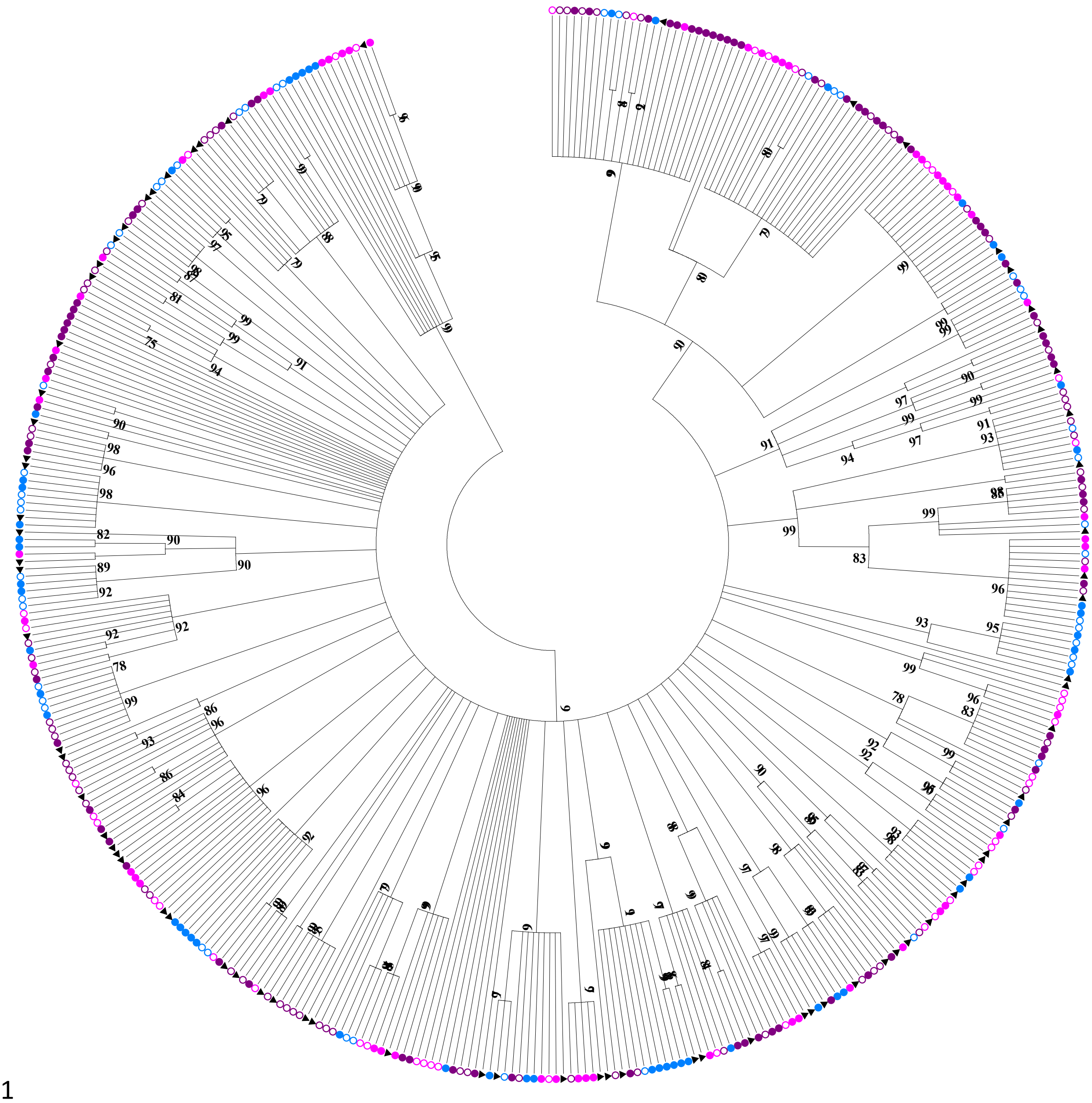

**Supplementary Figure 2: Phylogenetic analysis of HRV-A sequences**. South Africa(●), Mali(●) and Zambia(●) alongside reference strains from GenBank(▲). Sequences with closed circles are from cases and those with open circles were detected in controls. Bootstrap values after 1000 replicates are shown next to the branches, strains with nucleotide diversity <70% have been omitted from the tree. The phylogenetic tree is drawn to scale and the branch lengths are the same length of those used to infer the tree.
