# Supplementary material for: Molecular Subtyping of Human Rhinovirus in Children from Three Sub-Saharan African Countries
Source: J Clin Microbiol. 2019 Aug 26;57(9):e00723-19. doi: 10.1128/JCM.00723-19 (PMC6711929; doi:10.1128/JCM.00723-19)
Supplement: Supplemental file 5 [file JCM.00723-19-s0005.pdf]

Supplementary Table 1: The demographic and clinical characteristics of the RTI community controls infected with the three HRV species

Characteristics, n(%)	HRV-A (n=38)	HRV-B (n=11)	HRV-C (n=50)	Unadjusted P-value	aOR(95% CI)	Adjusted P-value
**Age in months, mean(SD)**	11.3 (7.6-14.9)	10.5 (3.9-17.2)	10.2 (7.9-12.6)	0.623		0.798
**Female**	19(50)	6(55)	21(42)	0.456	1.52(0.57-4.09)	0.402
**HIV+**	3(8)	1(9)	4(8)	0.296	1.14(0.41-3.22)	0.796
**HEU[a]**	5(14)	1(10)	3(7)	0.257	1.09(0.17-7.12)	0.928
**Never breast fed**	2(5)	1(9)	1(2)	0.421	2.12(0.15-30.1)	0.578
**Under weight[b]**	12(32)	2(18)	8(16)	0.089	2.58(0.87-7.62)	0.087
**Day care attendance**	15(40)	7(64)	29(58)	0.087	0.56(0.21-1.49)	0.247
**Smoker in household**	8(21)	7(9)	10(20)	0.903	1.0(0.30-3.37)	0.999
**Premature birth[c]**	5(13)	0	5(10)	0.909	0.68(0.16-2.83)	0.594
**Birth weight, mean(SD)**	2.9(2.8-3.2)	3.2(2.8-3.7)	3.0(2.8-3.2)	0.782		0.589
**<u>Clinical features:</u>**						
**Tachypnea[d]**	16(46)	5(50)	16(28)	0.163	1.04(0.33-3.20)	0.952
**Cough**	13(34)	2(18)	13(26)	0.512	1.41(0.50-3.93)	0.517
**Fever[e]**	1(3)	0	3(6)	0.562	0.27(0.02-4.14)	0.351
**Diarrhoea**	1(3)	1(9)	0	0.144		0.322
**Rhinorrhoea**	18(47)	4(36)	33(66)	0.087	0.83(0.29-2.36)	0.721
**<u>Laboratory markers:</u>**						
**CRP≥40mg/l[f]**	1(3)	0	1(2)	0.844	0.60(0.28-12.7)	0.746
***LytA* positive[g]**	5(13)	1(9)	0	0.034		0.089
**HDP[h]**						
**-Blood**	4(11)	1(9)	0	0.067		0.099
**-NP**	10(26)	1(9)	19(38)	0.250	0.73(0.26-2.04)	0.551
**HRV viral load, mean(SD)[i]**	3.3(2.9-3.6)		3.3(3.0-3.5)	0.983		0.959
**HRV mono-infection[j]**	22(58)	8(73)	28(56)	0.859	0.63(0.20-2.01)	0.440
**<u>Viral co-infections in the nasopharynx:</u>**						
**-AdV**	5(13)	0	7(14)	0.909	0.69(0.12-3.88	0.678
**-RSV**	1(3)	0	1(2)	0.844	1.16(0.51-26.8)	0.922
**-HBoV**	7(18)	2(18)	10(20)	0.853	1.03(0.31-3.51)	0.954
**-HMPV**	0	1(9)	0			
**-InFV A-C**	0	0	2(4)			
**-PIVs**	4(11)	0	2(4)	0.246	4.04(0.55-29.6)	0.168
**-HCoVs**	3(8)	0	9(18)	0.182	0.66(0.15-2.98)	0.592
**<u>Bacterial co-infections in the nasopharynx:</u>**						
**-S.pneumoniae**	31(82)	9(82)	41(82)	0.960	1.02(0.26-4.04)	0.972
**-S.aureus**	8(21)	3(27)	4(8)	0.087	2.64(0.66-10.6)	0.171
**-M.catarrhalis**	31(82)	9(82)	44(88)	0.404	0.60(0.16-2.25)	0.454
**-H.influenzae**	24(63)	5(45)	28(56)	0.499	1.59(0.58-4.39)	0.372

Abbreviations - HIV: human immunodeficiency virus; HEU: HIV exposed but uninfected; OR: odds ratio; aOR: adjusted odds ratio; CI: confidence interval; SD: standard deviation; HDP: high density pneumococcus; CRP: C-reactive protein; NP: nasopharyngeal; HRV: Human rhinovirus; RSV: Respiratory Syncytial Virus(A and B), HMPV: Human Metapneumovirus; AdV: Adenovirus; PIV: Parainfluenza type 1-4; HBoV: Human Bocavirus; HCoV: Coronavirus(OC43, NL63, 229E and HKU1); InFV A-C: Influenza Virus(A, B and C)); *S.pneumoniae: Streptococcus pneumoniae; S.aureus: Staphylococcus pneumoniae; M.catarrhalis: Moraxella catarrhalis and H.influenzae: Haemophilus influenza.*

P-values calculated by comparing HRV-A to HRV-C using Chi-square and Wilcoxon tests - logistic regression models adjusted for confounding variates(*P*-values<0.2 in univariate analysis) where applicable; Odds ratios could not be calculated for variables with zero variables.

a - HEU defined as HIV-uninfected but HIV-exposed. Undetectable viral load, HIV seronegative in the child with a positive maternal history. Positive maternal status based on self-report was accepted, except for seronegative children <7 months of age where documented positive maternal status was required;

b - Underweight defined as weight for age <-2SD of the median age-sex specific WHO reference;

c - Premature birth defined as gestational age <37 weeks;

d - Tachypnea defined as respiratory rate >60 breaths/minute if aged <2 months, respiratory rate >50 breaths/minute if aged 2-12 months, respiratory rate >40 breaths/minute if aged >12 months;

e - Fever defined as temperature ≥38°C;

f - CRP defined as levels ≥40 mg/L which are considered to show potential bacterial infection. Only a subset of randomly chosen controls had CRP testing conducted at the South African site;

g - Blood sample positive for *S. pneumoniae* colonisation by *LytA* PCR;

h - HDP defined as *S. pneumoniae* density in nasopharynx >6.9 log10 copies/mL and/or density in whole blood sample >2.2 log10 copies/mL;

i - HRV viral load in the nasopharynx, expressed as log10 copies/mL;

j - HRV was the only respiratory virus detected in the nasopharynx.
