# Supplementary material for: Molecular Subtyping of Human Rhinovirus in Children from Three Sub-Saharan African Countries
Source: J Clin Microbiol. 2019 Aug 26;57(9):e00723-19. doi: 10.1128/JCM.00723-19 (PMC6711929; doi:10.1128/JCM.00723-19)
Supplement: Supplemental file 6 [file JCM.00723-19-s0006.pdf]

Supplementary Table 2: The demographic and clinical characteristics of the asymptomatic community controls infected with the three HRV species

Characteristics, n(%)	HRV-A (n=33)	HRV-B (n=5)	HRV-C((n=48)	Unadjusted P-value	aOR(95% CI)	Adjusted P-value
**Age in months, mean(SD)**	9.3(7.8-10.8)	6(3.6-8.4)	11.0(9.1-12.9)	0.167		0.162
**Female**	74(49)	17(59)	68(48)	0.938	0.99(0.61-1.62)	0.976
**HIV+**	11(7)	0	9(6)	0.523	0.55(0.28-1.08)	0.083
**HEU**[a]	27(19)	5(17)	36(27)	0.088	0.67(0.38-1.17)	0.158
**Never breast fed**	36(24)	4(14)	30(21)	0.622	1.72(0.86-3.46)	0.125
**Under weight**[b]	10(7)	5(17)	8(6)	0.747	1.42(0.52-3.91)	0.494
**Day care attendance**	29(19)	7(24)	36(26)	0.296	0.67(0.38-1.19)	0.175
**Smoker in household**	45(30)	5(17)	42(30)	0.973	0.80(0.46-1.37)	0.415
**Premature birth**[c]	29(19)	6(21)	27(19)	0.919	1.33(0.71-2.49)	0.377
**Birth weight, mean(SD)**	3.0(2.9-3.1)	2.9(2.6-3.1)	3.1(2.9-3.1)	0.197		0.161
**<u>Laboratory markers:</u>**						
**CRP≥40mg/l**[d]	1(1)	0	1(1)	0.957		0.998
***LytA* positive**[e]	14(9)	3(10)	7(5)	0.171	2.26(0.85-6.0)	0.101
**HDP**[f]						
**-Blood**	8(5)	2(7)	5(4)	0.488	1.44(0.43-4.86)	0.560
**-NP**	39(26)	3(10)	32(23)	0.554	1.10(0.64-1.91)	0.727
**HRV viral load, mean(SD)**[g]	3.6(3.4-3.7)	3.4(3.1-3.7)	3.7(3.5-3.8)	0.345		0.426
**HRV mono-infection**[h]	108(71)	19(66)	95(67)	0.496	1.17(0.59-2.30)	0.652
**<u>Viral co-infections in the nasopharynx:</u>**						
**-AdV**	12(8)	3(10)	15(11)	0.419	0.72(0.31-1.70)	0.455
**-RSV**	4(3)	0	4(3)	0.914	0.93(0.22-3.87)	0.919
**-HBoV**	14(9)	2(7)	15(11)	0.683	0.95(0.40-2.26)	0.901
**-HMPV**	4(3)	1(3)	0	0.052		0.587
**-InFV A-C**	1(1)	0	2(1)	0.528	0.57(0.05-7.08)	0.665
**-PIVs**	8(5)	2(7)	2(1)	0.091	2.31(0.45-11.89)	0.316
**-HCoVs**	13(9)	3(10)	20(14)	0.131	0.56(0.56-1.22)	0.144
**<u>Bacterial co-infections in the nasopharynx:</u>**						
**-S.pneumoniae**	119(78)	18(62)	115(82)	0.486	0.81(0.44-1.50)	0.504
**-S.aureus**	22(14)	9(31)	17(12)	0.543	1.25(0.58-2.68)	0.571
**-M.catarrhalis**	108(71)	19(66)	109(77)	0.223	0.72(0.40-1.26)	0.249
**-H.influenzae**	80(53)	11(38)	68(48)	0.451	1.29(0.79-2.11)	0.308

Abbreviations - HIV: human immunodeficiency virus; HEU: HIV exposed but uninfected; OR: odds ratio; aOR: adjusted odds ratio; CI: confidence interval; SD: standard deviation; HDP: high density pneumococcus; CRP: C-reactive protein; NP: nasopharyngeal; HRV: Human rhinovirus; RSV: Respiratory Syncytial Virus(A and B), HMPV: Human Metapneumovirus; AdV: Adenovirus; PIV: Parainfluenza type 1-4; HBoV: Human Bocavirus; HCoV: Human Coronavirus(OC43, NL63, 229E and HKU1); InFV A-C: Influenza Virus(A, B and C)); *S.pneumoniae: Streptococcus pneumoniae; S.aureus: Staphylococcus pneumoniae; M.catarrhalis: Moraxella catarrhalis and H.influenzae: Haemophilus influenza.*

P-values calculated by comparing HRV-A to HRV-C using Chi-square and Wilcoxon tests - logistic regression models adjusted for confounding variates(*P*-values<0.2 in univariate analysis) where applicable; Odds ratios could not be calculated for variables with zero variables.

a - HEU defined as HIV-uninfected but HIV-exposed. Undetectable viral load, HIV seronegative in the child with a positive maternal history. Positive maternal status based on self-report was accepted, except for seronegative children <7 months of age where documented positive maternal status was required;

b - Underweight defined as weight for age <-2SD of the median age-sex specific WHO reference;

c - Premature birth defined as gestational age <37 weeks;

d - CRP defined as levels ≥40 mg/L which are considered to show potential bacterial infection. Only a subset of randomly chosen controls had CRP testing conducted at the South African site;

e - Blood sample positive for *S. pneumoniae* colonisation by *LytA* PCR;

f - HDP defined as *S. pneumoniae* density in nasopharynx >6.9 log10 copies/mL and/or density in whole blood sample >2.2 log10 copies/mL;

g - HRV viral load in the nasopharynx, expressed as log10 copies/mL;

h - HRV was the only respiratory virus detected in the nasopharynx.
